# Association of the *LIPG *584C > T polymorphism and serum lipid levels in the Guangxi Bai Ku Yao and Han populations

**DOI:** 10.1186/1476-511X-9-110

**Published:** 2010-10-06

**Authors:** Wan-Ying Liu, Rui-Xing Yin, Lin Zhang, Xiao-Li Cao, Lin Miao, Dong-Feng Wu, Lynn Htet Htet Aung, Xi-Jiang Hu, Wei-Xiong Lin, De-Zhai Yang

**Affiliations:** 1 Department of Cardiology, Institute of Cardiovascular Diseases, the First Affiliated Hospital, Guangxi Medical University, 22 Shuangyong Road, Nanning 530021, Guangxi, People's Republic of China; 2 Department of Molecular Biology, Medical Scientific Research Center, Guangxi Medical University, 22 Shuangyong Road, Nanning 530021, Guangxi, People's Republic of China

## Abstract

**Background:**

Endothelial lipase (EL) is a major determinant of high-density lipoprotein-cholesterol (HDL-C) metabolism, but the association of endothelial lipase gene (*LIPG*) polymorphism and serum HDL-C levels is scarce and conflicting in diverse populations. Bai Ku Yao is an isolated subgroup of the Yao minority in China. This study was designed to detect the association of *LIPG *584C > T (rs2000813) polymorphism and several environmental factors with serum lipid levels in the Guangxi Bai Ku Yao and Han populations.

**Methods:**

A total of 645 subjects of Bai Ku Yao and 638 participants of Han Chinese were randomly selected from our previous stratified randomized cluster samples. Genotyping of the *LIPG *584C > T was performed by polymerase chain reaction and restriction fragment length polymorphism combined with gel electrophoresis, and then confirmed by direct sequencing.

**Results:**

The levels of serum total cholesterol (TC), HDL-C, low-density lipoprotein cholesterol (LDL-C) and apolipoprotein (Apo) AI and ApoB were lower in Bai Ku Yao than in Han (*P *< 0.05 - 0.001). The frequency of C and T alleles was 73.5% and 26.5% in Bai Ku Yao, and 67.9% and 32.1% in Han (*P *< 0.01); respectively. The frequency of CC, CT and TT genotypes was 50.4%, 46.2% and 3.4% in Bai Ku Yao, and 41.4%, 53.1% and 5.5% in Han (*P *< 0.01); respectively. Serum HDL-C levels in both ethnic groups were different among the three genotypes (*P *< 0.05 for each). Serum TC levels in both ethnic groups were also different between the CC and CT/TT genotypes (*P *< 0.05 for each). The T allele carriers had higher serum HDL-C and TC levels than the T allele noncarriers. Multivariate logistic regression analysis showed that the levels of HDL-C and ApoB were correlated with genotypes in Bai Ku Yao (*P *< 0.05 for each), whereas the levels of TC and HDL-C were associated with genotypes in Han Chinese (*P *< 0.05 and *P *< 0.01). Serum lipid parameters were also correlated with several environmental factors in the both ethnic groups.

**Conclusions:**

The frequency of *LIPG *584T allele is lower in Bai Ku Yao than in Han Chinese. The *LIPG *584T allele is associated with increased serum HDL-C, TC and ApoB levels. The differences in serum HDL-C, TC and ApoB levels between the two ethnic groups might partly result from different genotypic and allelic frequencies of *LIPG *584C > T or different *LIPG*-enviromental interactions.

## Introduction

Many epidemiological and clinical studies have shown that dyslipidemia, including high levels of plasma or serum total cholesterol (TC) [1,2], triglyceride (TG) [3,4], low-density lipoprotein cholesterol (LDL-C) [5,6] and apolipoprotein B (ApoB) [7,8], and low levels of high-density lipoprotein cholesterol (HDL-C) and ApoAI [9,10], is strongly associated with an increased risk of coronary artery disease (CAD). It is generally accepted that dyslipidemia is a polygenic disease with pathogenic contributions from both genetic and environmental risk factors such as demographics [11], diet [12], alcohol consumption [13], cigarette smoking [13,14], obesity [15], physical activity [16], hypertension [17]. Data from family and twin studies suggest that genetic variation accounts for 40%-60% of the individual variation in serum lipid concentrations [18-20].

Endothelial lipase (protein: EL; gene: *LIPG*) is a member of the triglyceride lipase family of proteins that includes lipoprotein lipase and hepatic lipase, and it exhibits a conserved catalytic triad, heparin-binding properties, lipid-binding domains, and cysteine residues [21]. EL is produced by endothelial cells as well as by other cell types such as macrophages and hepatocytes. *LIPG *spans 10 exons and 9 introns and encodes a polypeptide of 500 amino acids. The EL cDNA shares 45% homology with lipoprotein lipase and 40% homology with hepatic lipase and contains three conserved catalytic residues [21,22]. EL has phospholipase activity, but relatively little triglyceride lipase activity (phospholipase to triglyceride lipase ratio, 1.6) [23-25]. It has been demonstrated that overexpression of EL in the liver by adenovirus-mediated gene transfer results in a significant decrease in HDL-C and ApoAI [21]. Among the genetic variants of the *LIPG*, a common single nucleotide polymorphism (SNP), 584C > T, in exon 3 deserved greater scrutiny, as it was responsible for a significant amino acid change (584C > T, Thr111Ile) that could potentially be associated with altered EL activity. Studies in mouse models showed that a decrease in EL expression and activity, by gene deletion of *LIPG *in knockout mice [26,27] and by antibody inhibition [28], resulted in significant increases in plasma HDL-C in mice. Furthermore, overexpression of *LIPG *in transgenic mice resulted in decreased plasma HDL [21]. The *LIPG *584C > T (rs2000813) polymorphism in humans has been found to be associated with modifications of serum HDL-C levels in some studies [27,29-32] but not in others [33-37].

Han is the largest ethnic group and Yao is the eleventh largest minority among the 55 minority groups according to the population size. Bai Ku Yao (White-trouser Yao), an isolated ethnic subgroup of the Yao minority, is named so because all the men wear white knee-length knickerbockers. The population size is about 30000. Because of isolation from the other ethnic groups, the special customs and cultures including their clothing, intra-ethnic marriages, dietary habits, and lifestyle are still completely preserved to the present day. In several previous epidemiologic study, we found that the serum lipid levels and the prevalence of hyperlipidemia were lower in Bai Ku Yao than in Han Chinese from the same area [38,39]. This ethnic difference in serum lipid profiles is still not well known. We hypothesized that some gene polymorphisms may be different between the two ethnic groups. Therefore, in the present study, we examined the associations of *LIPG *584C > T polymorphism and several environmental factors with serum lipid levels in the Guangxi Bai Ku Yao and Han populations.

## Materials and methods

### Study population

A total of 645 subjects of Bai Ku Yao who reside in Lihu and Baxu villages in Nandan County, Guangxi Zhuang Autonomous Region, People's Republic of China were randomly selected from our previous stratified randomized cluster samples [38,39]. The ages of the subjects ranged from 15 to 85 years, with an average age of 40.25 ± 15.28 years. There were 337 males (52.25%) and 308 females (47.75%). All subjects were rural agricultural workers. The subjects accounted for 2.15% of total Bai Ku Yao population. During the same period, a total of 638 people of Han Chinese who reside in the same villages were also randomly selected from our previous stratified randomized cluster samples [38,39]. The mean age of the subjects was 40.53 ± 15.54 years (range 15 to 82). There were 343 men (53.76%) and 295 women (46.24%). All of them were also rural agricultural workers. All study subjects had no evidence of any chronic illness, including hepatic, renal, or thyroid. The participants with a history of heart attack or myocardial infarction, stroke, congestive heart failure, diabetes or fasting blood glucose ≥7.0 mmol/L determined by glucose meter have been excluded. The participants were not taking medications known to affect serum lipid levels (lipid-lowering drugs such as statins or fibrates, beta-blockers, diuretics, or hormones). The present study was approved by the Ethics Committee of the First Affiliated Hospital, Guangxi Medical University. Informed consent was obtained from all subjects after they received a full explanation of the study.

### Epidemiological survey

The survey was carried out using internationally standardized methods, following a common protocol [40]. Information on demographics, socioeconomic status, and lifestyle factors was collected with standardized questionnaires. The alcohol information included questions about the number of liangs (about 50 g) of rice wine, corn wine, rum, beer, or liquor consumed during the preceding 12 months. Alcohol consumption was categorized into groups of grams of alcohol per day: <25 and ≥25. Smoking status was categorized into groups of cigarettes per day: <20 and ≥20. At the physical examination, several anthropometric parameters, such as height, weight, and waist circumference were measured. Sitting blood pressure was measured three times with the use of a mercury sphygmomanometer after the subjects had a 5-minute rest, and the average of the three measurements was used for the blood pressure levels. Systolic blood pressure was determined by the first Korotkoff sound, and diastolic blood pressure by the fifth Korotkoff sound. Body weight, to the nearest 50 grams, was measured using a portable balance scale. Subjects were weighed without shoes and in a minimum of clothing. Height was measured, to the nearest 0.5 cm, using a portable steel measuring device. From these two measurements body mass index (BMI, kg/m^2^) was calculated.

### Laboratory methods

A venous blood sample of 8 ml was obtained from all subjects between 8 and 11 AM, after at least 12 hours of fasting, from a forearm vein after venous occlusion for few seconds in a sitting position. A part of the sample (3 ml) was collected into glass tube and used to determine serum lipid levels. Another part of the sample (5 ml) was transferred to tubes with anticoagulate solution (4.80 g/L citric acid, 14.70 g/L glucose, and 13.20 g/L tri-sodium citrate) and used to extract DNA. Immediately following clotting serum was separated by centrifugation for 15 minutes at 3000 rpm. The levels of TC, TG, HDL-C, and LDL-C in samples were determined by enzymatic methods with commercially available kits, Tcho-1, TG-LH (RANDOX Laboratories Ltd., Ardmore, Diamond Road, Crumlin Co. Antrim, United Kingdom, BT29 4QY), Cholestest N HDL, and Cholestest LDL (Daiichi Pure Chemicals Co., Ltd., Tokyo, Japan), respectively. Serum ApoAI and ApoB levels were detected by the immunoturbidimetric immunoassay using a commercial kit (RANDOX Laboratories Ltd.). All determinations were performed with an autoanalyzer (Type 7170A; Hitachi Ltd., Tokyo, Japan) in the Clinical Science Experiment Center of the First Affiliated Hospital, Guangxi Medical University [38,39].

### Genetic analysis

Genomic DNA was isolated from peripheral blood leukocytes using the phenol-chloroform method [41,42]. The extracted DNA was stored at 4°C until analysis. Genotyping of the *LIPG *584C > T was performed by polymerase chain reaction and restriction fragment length polymorphism (PCR-RFLP) [37]. *LIPG *genotypes were determined using mutagenic oligonucleotide primers with sequences 5'-CATGAGCTGAGATTGTTGTCAGTGC-3' and 5'-CAGTCAACCACAACTACATTGGCGTCTTTCTCTCAT-3' (Sangon, Shanghai, People's Republic of China). Each amplification reaction was performed in a total volume of 25 mL, containing 10 × PCR buffer (1.8 mM MgCl_2_) 2.5 μL, 1 U *Taq *polymerase, 2.5 mmol/L of each dNTP (Tiangen, Beijing, People's Republic of China) 2.0 μL, 20 pmol/L of each primer and 50 ng of genomic DNA, processing started with 94°C for 5 min and 30 cycles at 94°C for 30 s, 58.8°C for 30 s and 72°C for 30 s. This was followed by a final extension at 72°C for 4 min. Then 10 U of *Nde*I enzyme was added directly to the PCR products (10 μL) and digested at 37°C overnight. After restriction enzyme digestion of the amplified DNA, genotypes were identified by electrophoresis on 3% agarose gels and visualized with ethidium-bromide staining ultraviolet illumination. Genotypes were scored by an experienced reader blinded to epidemiological and serum lipid data. Six samples (CC, CT and TT genotypes in two, respectively) detected by the PCR-RFLP were also confirmed by direct sequencing. The PCR product was purified by low melting point gel electrophoresis and phenol extraction, and then the DNA sequence was analyzed in Shanghai Sangon Biological Engineering Technology & Services Co., Ltd., People's Republic of China.

### Diagnostic criteria

The normal values of serum TC, TG, HDL-C, LDL-C, ApoAI and ApoB levels, and the ratio of ApoAI to ApoB in our Clinical Science Experiment Center were 3.10-5.17, 0.56-1.70, 0.91-1.81, 2.70-3.20 mmol/L, 1.00-1.78, 0.63-1.14 g/L, and 1.00-2.50; respectively. The individuals with TC > 5.17 mmol/L and/or TG > 1.70 mmol/L were defined as hyperlipidemic [38,39]. Hypertension was diagnosed according to the criteria of 1999 World Health Organization-International Society of Hypertension Guidelines for the management of hypertension [43,44]. The diagnostic criteria of overweight and obesity were according to the Cooperative Meta-analysis Group of China Obesity Task Force. Normal weight, overweight and obesity were defined as a BMI <24, 24-28, and > 28 kg/m^2^; respectively [45].

### Statistical analyses

Epidemiological data were recorded on a pre-designed form and managed with Excel software. All statistical analyses were done with the statistical software package SPSS 13.0 (SPSS Inc., Chicago, Illinois). Quantitative variables were expressed as mean ± standard deviation (serum TG levels were presented as medians and interquartile ranges). Qualitative variables were presented as percentages. Allele frequency was determined via direct counting, and the standard goodness-of-fit test was used to test the Hardy-Weinberg equilibrium. Differences in genotype distribution between the ethnic groups were obtained using the chi-square test. The difference in general characteristics between Bai Ku Yao and Han Chinese was tested by the Student's unpaired *t*-test (TG by the Wilcoxon-Mann-Whitney test). The association of genotypes with serum lipid parameters was tested by analysis of covariance (TG by the Kruskal-Wallis test or the Wilcoxon-Mann-Whitney test). Sex, age, BMI, blood pressure, alcohol intake, cigarette smoking were adjusted for the statistical analysis. In order to evaluate the association of serum lipid levels with genotypes and several environment factors, multivariate logistic regression analysis was also performed in the combined population of Bai Ku Yao and Han, Bai Ku Yao, and Han; respectively. A *P *value of less than 0.05 was considered statistically significant.

## Results

### General characteristics and serum lipid levels

Table 1 gives the general characteristics and serum lipid levels between the Bai Ku Yao and Han populations. The levels of body height, weight, BMI, systolic blood pressure, diastolic blood pressure, pulse pressure, serum TC, HDL-C, LDL-C, ApoAI, and ApoB were lower in Bai Ku Yao than in Han (*P *< 0.05 - 0.001), whereas the percentage of subjects who consumed alcohol was higher in Bai Ku Yao than in Han (*P *< 0.05). There was no significant difference in serum TG levels, age structure, the percentage of subjects who smoked cigarettes, and the ratio of ApoAI to ApoB, or the ratio of male to female between the two ethnic groups (*P *> 0.05).

**Table 1:** The general characteristics and serum lipid levels between the Bai Ku Yao and Han populations

Parameter	Bai Ku Yao	Han Chinese	*t *(χ^2^)	*P*
Number	645	638	-	-
Male/female	337/308	343/295	0.295	0.587
Age (years)	40.25 ± 15.28	40.53 ± 15.54	-0.327	0.743
Height (cm)	153.04 ± 7.17	156.14 ± 7.97	-7.322	0.000
Weight (kg)	51.53 ± 7.15	54.83 ± 9.38	-7.079	0.000
Body mass index (kg/m^2^)	21.97 ± 2.37	22.45 ± 3.23	-3.080	0.002
Systolic blood pressure (mmHg)	117.03 ± 16.07	120.89 ± 16.45	-4.251	0.000
Diastolic blood pressure (mmHg)	74.92 ± 9.11	76.46 ± 10.77	-2.767	0.006
Pulse pressure (mmHg)	42.11 ± 11.96	44.47 ± 11.26	-3.632	0.000
Cigarette smoking [n (%)]				
Nonsmoker	451(69.9)	440(69.0)		
<20 cigarettes/day	88(13.6)	80(12.5)		
≥20 cigarettes/day	106(16.5)	118(18.5)	1.121	0.571
Alcohol consumption [n (%)]				
Nondrinker	363(56.3)	400(62.7)		
<25 g/day	183(28.4)	146(22.9)		
≥25 g/day	99(15.3)	92(14.4)	6.174	0.046
Total cholesterol (mmol/L)	4.28 ± 0.93	4.68 ± 1.05	-7.315	0.000
Triglyceride (mmol/L)^a^	1.02(0.71)	0.98(0.73)	-0.450	0.653
HDL-C (mmol/L)	1.62 ± 0.42	1.87 ± 0.49	-9.779	0.000
LDL-C (mmol/L)	2.51 ± 0.77	2.60 ± 0.77	-2.148	0.032
Apolipoprotein (Apo) AI (g/L)	1.28 ± 0.33	1.41 ± 0.27	-7.862	0.000
ApoB (g/L)	0.83 ± 0.23	0.88 ± 0.22	-4.115	0.000
ApoAI/ApoB	1.67 ± 0.77	1.70 ± 0.53	-0.575	0.565

HDL-C, high-density lipoprotein cholesterol; LDL-C, low-density lipoprotein cholesterol. ^a^The value of TG was presented as median (interquartile range). The difference between the two ethnic groups was determined by the Wilcoxon-Mann-Whitney test.

### Results of electrophoresis and genotyping

**Figure 1:**
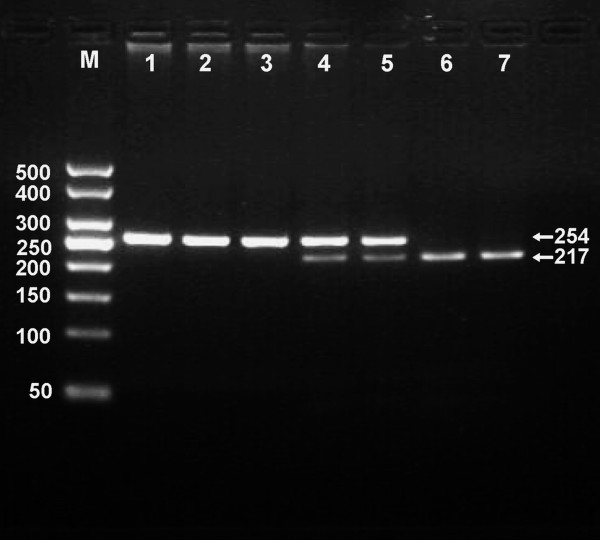
**Genotyping of the *LIPG *584C > T polymorphism**. Lane M, 50 bp Marker Ladder; lane 1, the PCR product of the sample (254 bp); lanes 2 and 3, CC genotype (254 bp); lanes 4 and 5, CT genotype (254-, 217- and 37-bp); and lanes 6 and 7, TT genotype (217- and 37-bp). The 37 bp fragment was invisible in the gel owing to its fast migration speed.

After the genomic DNA of the samples was amplified by PCR and imaged by 3% agarose gel electrophoresis, the purpose gene of 254 bp nucleotide sequences could be found in all samples (Figure 1). The genotypes identified were named according to the presence or absence of the enzyme restriction sites, when a C to T transversion at nucleotide position 584 of the *LIPG*. For the C allele, there was no site for *Nde*I; for the T allele, *Nde*I digestion produced 217- and 37-bp products. Thus, the CC genotype is homozygote for the absence of the site (band at 254 bp), CT genotype is heterozygote for the absence and presence of the site (bands at 254-, 217- and 37-bp), and TT genotype is homozygote for the presence the site (bands at 217- and 37-bp; Figure 1). The 37 bp fragment was invisible in the gel owing to its fast migration speed. The genotype distribution was consistent with the Hardy-Weinberg equilibrium.

### Genotypic and allelic frequencies

The frequencies of the *LIPG *584C > T alleles and genotypes are shown in Table 2. The frequencies of C and T alleles were 73.5% and 26.5% in Bai Ku Yao, and 67.9% and 32.1% in Han (*P *< 0.01); respectively. The frequencies of CC, CT and TT genotypes were 50.4%, 46.2% and 3.4% in Bai Ku Yao, and 41.4%, 53.1% and 5.5% in Han (*P *< 0.01); respectively.

**Table 2:** Genotypic and allelic frequencies of the *LIPG *584C > T polymorphism between the Bai Ku Yao and Han population [n (%)]

Group	n	Genotype			Allele	
		CC	CT	TT	C	T
Bai Ku Yao	645	325(50.4)	298(46.2)	22(3.4)	948(73.5)	342(26.5)
Han Chinese	638	264(41.4)	339(53.1)	35(5.5)	867(67.9)	409(32.1)
χ^2^	-	11.883			9.516	
*P*	-	0.003			0.002	

### Results of sequencing

**Figure 2:**
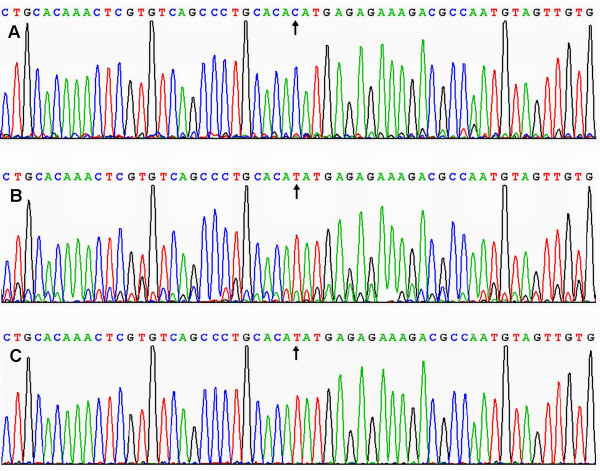
**A part of the nucleotide sequence of the *LIPG *584C > T polymorphism**. (A) CC genotype; (B) CT genotype; (C) TT genotype

The results shown as CC, CT and TT genotypes by PCR-RFLP, CC, CT and TT genotypes were also confirmed by sequencing (Figure 2).

### Genotypes and serum lipid levels

As shown in Table 3, the levels of HDL-C in both ethnic groups were different among the three genotypes (*P *< 0.05 for each). The levels of TC, HDL-C and ApoAI in Bai Ku Yao and the levels of TC and TG in Han were different between CC and CT/TT genotypes (*P *< 0.05 for all). The T allele carriers had higher serum lipid levels than the T allele noncarriers. There was no significant difference in the levels of LDL-C, ApoB, and the ratio of ApoAI to ApoB among the three genotypes in the both ethnic groups (*P *> 0.05 for all).

**Table 3:** Genotypic frequencies of the *LIPG *584C > T and serum lipid levels between the Bai Ku Yao and Han populations

Genotype	n	TC (mmol/L)	TG (mmol/L)^a^	HDL-C (mmol/L)	LDL-C (mmol/L)	ApoAI (g/L)	ApoB (g/L)	ApoAI/ApoB
Bai Ku Yao								
CC	325	4.20 ± 0.87	1.02(0.65)	1.58 ± 0.40	2.48 ± 0.71	1.25 ± 0.32	0.82 ± 0.22	1.63 ± 0.70
CT	298	4.34 ± 1.00	1.02(0.77)	1.66 ± 0.45	2.52 ± 0.83	1.30 ± 0.34	0.83 ± 0.23	1.71 ± 0.83
TT	22	4.51 ± 0.71	1.08(0.70)	1.78 ± 0.41	2.58 ± 0.63	1.40 ± 0.37	0.88 ± 0.25	1.76 ± 0.88
*F*	-	2.114	1.160	3.318	0.171	2.536	0.381	0.776
*P*	-	0.122	0.314	0.037	0.843	0.080	0.683	0.461
CC	325	4.20 ± 0.87	1.02(0.65)	1.58 ± 0.40	2.48 ± 0.71	1.25 ± 0.32	0.82 ± 0.22	1.63 ± 0.70
CT/TT	320	4.35 ± 0.98	1.03(0.77)	1.67 ± 0.45	2.53 ± 0.82	1.31 ± 0.34	0.83 ± 0.23	1.71 ± 0.83
*F*	-	3.894	1.774	5.414	0.310	3.902	0.119	1.522
*P*	-	0.049	0.183	0.020	0.578	0.049	0.731	0.218
Han Chinese								
CC	264	4.57 ± 0.88	0.99(0.64)	1.85 ± 0.48	2.54 ± 0.71	1.39 ± 0.25	0.87 ± 0.21	1.70 ± 0.50
CT	339	4.77 ± 1.18	0.97(0.78)	1.88 ± 0.48	2.65 ± 0.82	1.42 ± 0.29	0.89 ± 0.22	1.69 ± 0.56
TT	35	4.75 ± 0.91	1.08(0.83)	2.09 ± 0.63	2.49 ± 0.67	1.46 ± 0.23	0.91 ± 0.22	1.70 ± 0.56
*F*	-	2.959	2.781	3.904	1.951	1.549	1.215	0.012
*P*	-	0.053	0.063	0.021	0.143	0.213	0.297	0.988
CC	264	4.57 ± 0.88	0.99(0.64)	1.85 ± 0.48	2.54 ± 0.71	1.39 ± 0.25	0.87 ± 0.21	1.70 ± 0.50
CT/TT	374	4.76 ± 1.15	0.98(0.79)	1.89 ± 0.50	2.63 ± 0.80	1.43 ± 0.28	0.89 ± 0.22	1.69 ± 0.56
*F*	-	5.894	4.462	1.880	2.399	2.559	2.090	0.743
*P*	-	0.015	0.035	0.171	0.122	0.110	0.149	0.389

TC, total cholesterol; TG, triglyceride; HDL-C, high-density lipoprotein cholesterol; LDL-C, low-density lipoprotein cholesterol; ApoAI, apolipoprotein AI; ApoB, apolipoprotein B; ApoAI/ApoB, the ratio of apolipoprotein AI to apolipoprotein B. ^a ^The value of TG was presented as median (interquartile range). The difference among the genotypes was determined by the Kruskal-Wallis test or the Wilcoxon-Mann-Whitney test.

### Risk factors for the lipid parameters

Multivariate logistic regression analysis showed that the levels of HDL-C and ApoB were correlated with genotypes in Bai Ku Yao (*P *< 0.05 for each), whereas the levels of TC and HDL-C were associated with genotypes in Han Chinese (*P *< 0.05 and < 0.01; respectively). Serum lipid parameters were also correlated with sex, age, weight, BMI, alcohol consumption, cigarette smoking, and blood pressure in the both ethnic groups (Table 4).

**Table 4:** Correlative factors for the lipid paramerers between the Bai Ku Yao and Han populations

Lipid	Relative factor	Regression coefficient	Standard error	χ^2^	*P*	Odds ratio	95%CI
Bai Ku Yao and Han							
TC	Ethnic group	-0.559	0.149	14.085	0.000	0.572	0.427-0.766
	Age	0.020	0.005	14.060	0.000	1.020	1.010-1.031
	Body mass index	0.155	0.026	35.363	0.000	1.167	1.109-1.229
	Systolic blood pressure	0.020	0.008	7.225	0.007	1.021	1.006-1.036
	Pulse pressure	-0.030	0.010	8.287	0.004	0.971	0.951-0.991
	Alcohol consumption	0.210	0.096	4.780	0.029	1.234	1.022-1.490
TG	Sex	-0.665	0.182	13.347	0.000	0.514	0.360-0.735
	Body mass index	0.169	0.027	40.311	0.000	1.184	1.124-1.247
	Alcohol consumption	0.229	0.109	4.428	0.035	1.258	1.016-1.558
	Genotype	0.354	0.158	4.996	0.025	1.425	1.045-1.943
HDL-C	Ethnic group	1.221	0.333	13.414	0.000	3.390	1.764-6.515
	Sex	-1.209	0.346	12.244	0.000	0.298	0.152-0.588
	Age	-0.037	0.012	9.899	0.002	0.964	0.942-0.986
	Alcohol consumption	-0.612	0.270	5.157	0.023	0.542	0.320-0.920
LDL-C	Age	0.025	0.006	15.978	0.000	1.026	1.013-1.039
	Body mass index	0.174	0.029	35.023	0.000	1.190	1.123-1.261
	Systolic blood pressure	0.020	0.009	5.034	0.025	1.020	1.003-1.038
	Alcohol consumption	-0.258	0.123	4.417	0.036	0.772	0.607-0.983
ApoAI	Ethnic group	1.450	0.142	104.456	0.000	4.264	3.229-5.631
	Sex	-0.720	0.154	21.860	0.000	0.487	0.360-0.658
	Age	-0.033	0.005	45.064	0.000	0.968	0.958-0.977
	Diastolic blood pressure	-0.022	0.008	8.463	0.004	0.978	0.964-0.993
	Alcohol consumption	-0.773	0.118	42.820	0.000	0.462	0.366-0.582
ApoB	Age	0.026	0.007	15.328	0.000	1.026	1.013-1.039
	Body mass index	0.175	0.030	33.906	0.000	1.191	1.123-1.263
	Pulse pressure	-0.025	0.012	4.047	0.044	0.976	0.952-0.999
ApoAI/ApoB	Ethnic group	0.704	0.181	15.128	0.000	2.022	1.418-2.884
	Systolic blood pressure	0.012	0.005	5.190	0.023	1.012	1.002-1.022
	Alcohol consumption	0.401	0.110	13.280	0.000	1.494	1.204-1.854
Bai Ku Yao							
TC	Age	0.029	0.008	14.523	0.000	1.030	1.014-1.045
	Weight	0.077	0.015	25.488	0.000	1.080	1.048-1.113
TG	Sex	-0.833	0.229	13.299	0.000	0.435	0.278-0.680
	Body mass index	0.197	0.045	19.055	0.000	1.218	1.115-1.331
HDL-C	Sex	-1.044	0.394	7.031	0.008	0.352	0.163-0.762
	Age	-0.033	0.014	5.814	0.016	0.968	0.943-0.994
	Genotype	-0.273	0.196	4.627	0.040	0.652	0.950-0.990
LDL-C	Age	0.031	0.009	13.469	0.000	1.032	1.015-1.049
	Weight	0.065	0.017	15.009	0.000	1.067	1.032-1.102
ApoAI	Sex	-0.546	0.208	6.898	0.009	0.579	0.386-0.871
	Age	-0.029	0.006	23.998	0.000	0.971	0.960-0.983
	Alcohol consumption	-0.800	0.151	27.987	0.000	0.449	0.334-0.604
ApoB	Body mass index	0.189	0.055	11.470	0.001	1.207	1.084-1.345
	Diastolic blood pressure	0.035	0.015	5.503	0.019	1.036	1.006-1.066
	Genotype	0.479	0.231	4.283	0.038	1.614	1.026-2.539
ApoAI/ApoB	Alcohol consumption	0.706	0.167	17.835	0.000	2.025	1.460-2.810
Han Chinese							
TC	Sex	0.775	0.227	11.633	0.001	2.170	1.390-3.386
	Age	0.019	0.007	7.576	0.006	1.019	1.006-1.033
	Weight	0.057	0.011	25.915	0.000	1.059	1.036-1.082
	Systolic blood pressure	0.022	0.010	5.340	0.021	1.022	1.003-1.042
	Pulse pressure	-0.035	0.014	6.611	0.010	0.966	0.940-0.992
	Alcohol consumption	0.286	0.140	4.150	0.042	1.331	1.011-1.751
	Genotype	0.327	0.148	4.886	0.027	1.387	1.038-1.854
TG	Weight	0.064	0.012	28.297	0.000	1.066	1.041-1.092
	Systolic blood pressure	0.015	0.007	4.789	0.029	1.015	1.002-1.029
	Cigarette smoking	0.318	0.132	5.855	0.016	1.375	1.062-1.779
HDL-C	Age	-0.062	0.023	7.056	0.008	0.940	0.898-0.984
	Genotype	-0.432	0.189	7.274	0.008	0.654	0.430-0.890
LDL-C	Age	0.025	0.008	10.053	0.002	1.026	1.010-1.042
	Body mass index	0.115	0.051	5.128	0.024	1.122	1.016-1.240
	Alcohol consumption	-0.449	0.177	6.438	0.011	0.638	0.451-0.903
ApoAI	Sex	-1.074	0.244	19.359	0.000	0.342	0.212-0.551
	Age	-0.043	0.008	27.185	0.000	0.958	0.942-0.973
	Diastolic blood pressure	-0.033	0.012	7.657	0.006	0.967	0.945-0.990
	Alcohol consumption	-0.558	0.186	8.973	0.003	0.572	0.397-0.825
ApoB	Age	0.030	0.008	13.584	0.000	1.030	1.014-1.047
	Body mass index	0.174	0.034	25.783	0.000	1.191	1.113-1.273
ApoAI/ApoB	Systolic blood pressure	0.022	0.008	7.514	0.006	1.022	1.006-1.038

TC, total cholesterol; TG, triglyceride; HDL-C, high-density lipoprotein cholesterol; LDL-C, low-density lipoprotein cholesterol; ApoAI, apolipoprotein AI; ApoB, apolipoprotein B; ApoAI/ApoB, the ratio of apolipoprotein AI to apolipoprotein B

## Discussion

The present study shows that the serum levels of TC, HDL-C, LDL-C, ApoAI and ApoB were lower in Bai Ku Yao than in Han Chinese. There were no significant differences in serum TG levels and the ratio of ApoAI to ApoB between the two ethnic groups. These findings are consistent with those of our previous epidemiological studies [38,39]. It is well known that dyslipidemia is a complex trait caused by multiple environmental and genetic factors and their interactions. Bai Ku Yao is a special subgroup of the Yao minority in China. Strict intra-ethnic marriages have been performed in this population from time immemorial. Therefore, we believed that the hereditary characteristics and some lipid metabolism-related gene polymorphisms in this population may be different from those in Han Chinese.

The frequency of the rare allele (584T) was found to be 10.3% in blacks, 31.2% in white controls, 32.6% in whites with high HDL-C [34], 26% in the Lipoprotein and Coronary Atherosclerosis Study (LCAS) population (white individuals including 27 or 7% African Americans)[27], 26% in Japanese [37], and 21.6% in healthy school-aged Japanese children [33]. In the present study, we showed that the frequency of the T allele and CT and TT genotypes was lower in Bai Ku Yao than in Han. The frequency of the T allele in Bai Ku Yao was also lower than that of previous studies in white controls [34]. These results indicate that the *LIPG *584C > T polymorphism may have significant difference in allele frequencies among diverse ethnics and between control subjects and those with high HDL-C levels. However, McCoy *et al. *[24] found that EL is completely inactive *in vitro *in the presence of serum, and deLemos *et al. *[34] found no significant difference in allele frequencies of six polymorphisms in the *LIPG *between individuals with normal and those with high HDL-C. Thus, the physiological role of EL in lipoprotein metabolism, if any, is still unknown.

The association between the *LIPG *584C > T polymorphism and plasma or serum lipid levels in humans has not been fully elucidated. In the present study, we found a significant association between the *LIPG *584C > T polymorphism and serum HDL-C and TC levels. In the both Bai Ku Yao and Han populations, we can detect an increased prevalence of the T allele in subjects with higher serum HDL-C and TC levels. Previous association studies of this SNP with plasma HDL-C levels produced conflicting results. Several studies have reported significant association between the *LIPG *584C > T polymorphism and HDL-C levels [27,29-32], whereas several reports failed to find a significant genetic effect on HDL-C concentrations [33-37]. Ma and colleagues [27] reported that the *LIPG *584C > T polymorphism was associated with an increase in HDL-C among 372 participants. The patients with the T T genotype have a 14% higher mean HDL-C compared with those with the CC genotype. Yamakawa-Kobayashi *et al*. [33] failed to detect an association of the *LIPG *584C > T with HDL-C levels in 340 Japanese children. Paradis and co-workers [46] found an association of the *LIPG *584C > T with increased levels of the HDL_3 _subfraction in 281 females. Halverstadt *et al*. [32] found an association of the *LIPG *584C > T with NMR measurements of HDL size in 83 healthy elderly participants but not with overall HDL-C levels. Mank-Seymour *et al*. [29] showed a weak association of the *LIPG *584C > T with increased HDL-C among 594 participants. Hutter *et al*. [30] found a weak association of the *LIPG *584C > T with HDL-C levels in 541 Japanese Americans. Tang *et al*. [47] found a weak association of the *LIPG *584C > T with increased HDL-C in 265 Chinese CAD cases and controls. The data by Edmondson *et al*. [36] from a combined sample of 3,845 participants, and functional studies of the variant definitively establish that the *LIPG *584C > T is not associated with HDL-C and *in vitro *studies show that it has normal lipolytic activity. The reason for these conflicting results is not yet known. The 584C > T SNP results in an amino acid change (from a polar to nonpolar amino acid) that occurs in a relatively poorly conserved area of the EL sequence, far from important sites encoding for the catalytic activity of defining the tertiary structure of the enzyme [48]. The high prevalence of the minor allele also suggests that it may not have a large effect on plasma HDL-C levels in the general population [46]. In a previous study, however, Shimizu *et al. *[37] found no significant association between the *LIPG *584C > T polymorphism and HDL-C levels, but multivariate regression analyses showed that the association of the *LIPG *584T allele with acute myocardial infarction was statistically significant and independent of other risk factors when age, sex, hypertension, hypercholesterolemia, and diabetes mellitus were included in the analyses. Thus, the *LIPG *584C > T polymorphism may be involved in the pathogenesis of acute myocardial infarction. In LCAS there was also a strong association of the *LIPG *584C > T polymorphism with the ratios of HDL-C/LDL-C and ApoAI/ApoB, and the mean plasma ApoCIII concentration [27]. In the present study, we also showed that the levels of TC and ApoAI in Bai Ku Yao and the levels of TC and TG in Han were different between the CC and CT/TT genotypes. The T allele carriers had higher serum lipid levels than the T allele noncarriers. In multivariate logistic regression analysis, the levels of HDL-C and ApoB were correlated with genotypes in Bai Ku Yao, whereas the levels of TC and HDL-C were associated with genotypes in Han Chinese. These results suggest that the *LIPG *584C > T polymorphism can also influence other serum lipid parameters except HDL-C in our populations.

As mentioned above, plasma lipid concentration is highly heritable but is also modifiable by environmental factors including demographics [11], diet [12], alcohol consumption [13], cigarette smoking [13,14], obesity [15], exercise [16], hypertension [17]. These factors could explain why the association between HDL-C concentration and the *LIPG *584C > T polymorphism was not observed in some previous studies. For example, heavy smokers have, on average, 9% lower HDL-C levels than matched nonsmokers [14]. Obesity is one of the most important factors in reducing HDL-C levels [15,49]. Evidences of association in different populations with different lifestyles and diet might suggest that the associations found are robust to a large number of genetic and environmental factors. In the present study, we found that many confounding factors affect serum lipid levels. Serum lipid parameters were correlated with age, sex, alcohol consumption, cigarette smoking, BMI, and blood pressure. These findings suggest that the environmental factors also play an important role in determing serum lipid levels in these populations. Differences in serum lipid levels between the two ethnic groups could be related to factors such as differences in the genetic background, dietary patterns and lifestyle factors and their interactions. Although Bai Ku Yao and Han reside in the same region, there was significant difference in their diet and lifestyle. Corn was the staple food and rice, soy, buckwheat, sweet potato, and pumpkin products were the subsidiary foods in Bai Ku Yao. Approximately 90% of the beverages were corn wine and rum. The alcohol content is about 15% (v/v). They are also accustomed to drink Hempseed soup. In contrast, rice was the staple food and corn, broomcorn, potato, and taro products were the subsidiary foods in Han. About 90% of the beverage was rice wine. The content of alcohol is about 30% (v/v). The staple and subsidiary foods are more favorable for serum lipid profiles in Bai Ku Yao than in Han. Corn contains abundant dietary fiber and plant protein. Dietary fiber can decrease serum TC levels [50]. Plant protein can promote the transportation and excretion of free cholesterol. Soy protein intake is effective in reducing TC by 9.3%, LDL-C by 12.9%, and TG by 10.5% and in increasing HDL-C by 2.4% [51]. Hypocholesterolemic activity of buckwheat protein product is far stronger than that of soy protein isolate [52]. Ludvik and his colleagues [53] found that ingestion of 4 g/day caiapo (the extract of white-skinned sweet potato) for 6 weeks reduces TC and LDL-C in type 2 diabetic patients previously treated by diet alone. Studies have demonstrated that pumpkin is a useful therapy for hypercholesterolemia through reducing oxidative stress and cholesterol levels [54]. A number of experimental and clinical studies have demonstrated that the beneficial effects of Hempseed or Hempseed oil on serum lipid profiles include: decreasing TC, TG and LDL-C levels [54,55], inhibiting lipid peroxidation [55], reducing atherogenic index [56], and increasing HDL-C levels [55,56].

## Conclusion

The results of the present study show that the *LIPG *584T allele frequency is lower in Bai Ku Yao than in Han Chinese. A significant association is found between the *LIPG *584C > T polymorphism and serum HDL-C and TC levels in the both ethnic groups. An increased prevalence of the 584T allele in subjects with higher serum HDL-C and TC levels is detected in the both populations. The levels of HDL-C and ApoB were correlated with genotypes in Bai Ku Yao, whereas the levels of TC and HDL-C were associated with genotypes in Han Chinese. The differences in serum HDL-C, TC and ApoB levels between the two ethnic groups might partly result from different *LIPG *584C > T polymorphism or different *LIPG*-enviromental interactions.

## Competing interests

The authors declare that they have no competing interests.

## Authors' contributions

WYL participated in the design, undertook genotyping, performed statistical analyses, and helped to draft the manuscript. RXY conceived the study, participated in the design, carried out the epidemiological survey, collected the samples, and drafted the manuscript. LZ, XLC, LM, DFW, LHHA and XJH collaborated to the genotyping. WXL and DZY carried out the epidemiological survey, collected the samples, and helped to carry out the genotyping. All authors read and approved the final manuscript.
